# Milk microbiome in dairy cattle and the challenges of low microbial biomass and exogenous contamination

**DOI:** 10.1186/s42523-021-00144-x

**Published:** 2021-11-18

**Authors:** Jolinda Pollock, Susannah J. Salter, Rebecca Nixon, Michael R. Hutchings

**Affiliations:** 1 SRUC Veterinary Services, Scotland’s Rural College, Edinburgh, UK; 2 Department of Veterinary Medicine, University of Cambridge, Cambridge, UK; 3 Animal and Veterinary Sciences, Scotland’s Rural College, Edinburgh, UK

## Abstract

**Background:**

The blanket usage of antimicrobials at the end of lactation (or “drying off”) in dairy cattle is under increasing scrutiny due to concerns about antimicrobial resistance. To lower antimicrobial usage in dairy farming, farmers are now encouraged to use “selective dry cow therapy” whereby only cows viewed as at high risk of mastitis are administered antimicrobial agents. It is important to gain a better understanding of how this practice affects the udder-associated microbiota and the potential knock-on effects on antimicrobial-resistant bacterial populations circulating on the farm. However, there are challenges associated with studying low biomass environments such as milk, due to known contamination effects on microbiome datasets. Here, we obtained milk samples from cattle at drying off and at calving to measure potential shifts in bacterial load and microbiota composition, with a critical assessment of contamination effects.

**Results:**

Several samples had no detectable 16S rRNA gene copies and crucially, exogenous contamination was detected in the initial microbiome dataset. The affected samples were removed from the final microbiome analysis, which compromised the experimental design and statistical analysis. There was no significant difference in bacterial load between treatments (P > 0.05), but load was lower at calving than at drying off (P = 0.039). *Escherichia coli* counts by both sequence and culture data increased significantly in the presence of reduced bacterial load and a decreasing trend of microbiome richness and diversity. The milk samples revealed diverse microbiomes not reflecting a typical infection profile and were largely comprised of gut- and skin-associated taxa, with the former decreasing somewhat after prolonged sealing of the teats.

**Conclusions:**

The drying off period had a key influence on microbiota composition and bacterial load, which appeared to be independent of antimicrobial usage. The interactions between drying off treatment protocol and milk microbiome dynamics are clearly complex, and our evaluations of these interactions were restricted by low biomass samples and contamination effects. Therefore, our analysis will inform the design of future studies to establish whether different selection protocols could be implemented to further minimise antimicrobial usage.

**Supplementary Information:**

The online version contains supplementary material available at 10.1186/s42523-021-00144-x.

## Introduction

Mastitis is a highly prevalent and economically detrimental disease affecting dairy cattle, with the cost of mastitis in the UK being estimated at £170 million per year [1, 2]. The drying off period marks the end of a lactation cycle, and the udder is commonly artificially sealed using commercially available teat sealants to enable healing and to minimise the potential for infection. However, new bacterial infections in the udder occur most frequently at drying off in comparison to any other time point during lactation [3], with new infections during the dry period occurring up to 10 times the rate of new infections during lactation [4]. Consequently, intramammary antimicrobials are commonly used at drying off to treat such infections.

Due to concerns about antimicrobial resistance, selective dry cow therapy is now being encouraged, whereby only cattle deemed most at risk of mastitis are administered long acting intramammary antimicrobials after lactation. This practice assumes that reduced antimicrobial usage will slow the development of antimicrobial-resistant bacteria. Somatic cell count readings obtained from milk samples are used as a proxy for the concentration of leucocytes in milk [5] and consequently used as a selection criterion for the appropriate drying off treatment protocol—using a teat sealant only, or a teat sealant and intramammary antimicrobials. Although selective dry cow therapy is viewed as a positive change in the industry, further work is required to establish how this practice affects bacterial populations and their abundance, and the potential knock-on effects on the antimicrobial resistance determinants in the udder.

Several studies have been carried out to investigate the milk microbiome in both healthy and infected udders [6–11], and the effects of dry cow therapy with or without antimicrobial agents [6]. There are well known challenges associated with studying low biomass microbial communities, primarily associated with contamination effects [12, 13]. Here, we use 16S rRNA gene sequencing, bacterial culture and quantitative PCR to assess changes in milk microbiome composition and bacterial load in cows between drying off and calving, with a critical assessment of contamination effects.

## Materials and methods

### Study farm and experimental design

The experiment was carried out at Langhill Dairy Farm in Midlothian, Scotland (Royal (Dick) School of Veterinary Studies, The University of Edinburgh). Ethical approval was obtained from the Royal (Dick) School of Veterinary Studies Veterinary Ethics Research Committee. On this unit, selective dry cow therapy is carried out routinely and individual somatic cell count (SCC) readings are used to establish the appropriate treatment (i.e., cows with higher SCC than 200,000 cells/ml are administered an intramammary antimicrobial).

**Fig. 1:**
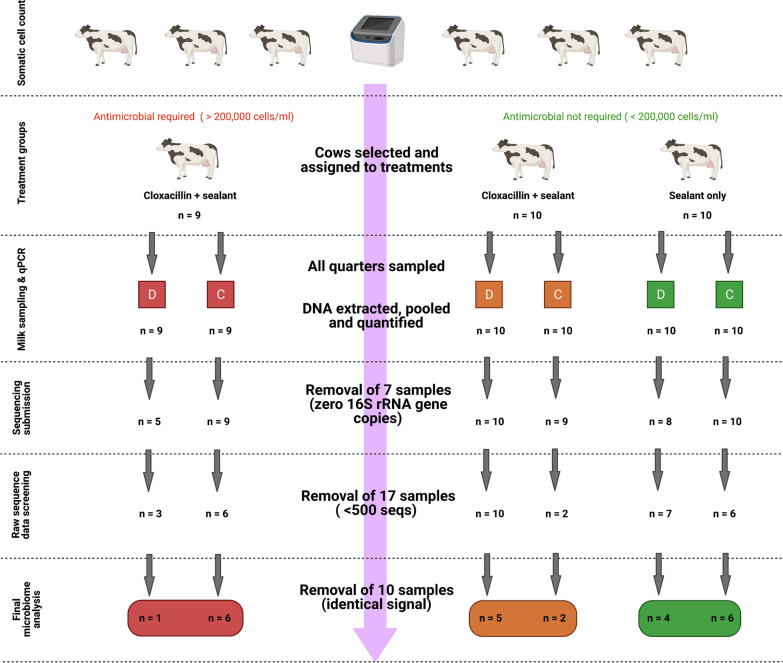
Experimental design and sample workflow from milk collection to the final 16S rRNA gene sequencing analysis. Samples included in the final analysis were subject to filtration based on 16S rRNA gene qPCR counts, 16S rRNA gene sequence counts and an exogenous contamination screen. Drying off = D, Calving = C. This Figure was created using BioRender.com

Prior to this study, cow identification numbers and pooled SCC readings were provided to allow selection of cattle for the experiment (n = 29). These Holstein–Friesian, multiparous cattle calved between December 2018 and February 2019. Using these data, cows were assigned to one of three treatment groups (high SCC with antimicrobial treatment (n = 9), and two low SCC groups with (n = 10) or without (n = 10) antimicrobial treatment) as outlined in Fig. 1. On animal welfare grounds, all cows with a SCC count higher than 200,000 cells/ml were administered the antimicrobial agent (cloxacillin) and teat sealant in each udder quarter. The mean somatic cell count for the high SCC group was 534,778 cells/ml, with the low SCC groups having means of 87,286 cells/ml (sealant only) and 76,182 cells/ml (sealant and antimicrobial). All cattle were housed in the same barn.

### Sample collection and DNA extraction

Two sets of milk samples were taken from each quarter of every cow—at drying off and immediately post-calving (Fig. 1). Before milk sampling, each teat was thoroughly cleaned using a standardised protocol by experienced farm staff [14]. Samples were drawn from each quarter and collected in 30 ml universal tubes and placed on wet ice for transportation. DNA extractions were immediately carried out in random order on 500 μl of fresh milk using the DNeasy PowerSoil Kit (Qiagen, UK) following the manufacturer’s instructions. A DNA pool for each cow per sampling point was then created by combining an equal volume of DNA extract from each quarter, with each cow/udder being classified as the experiment unit. DNA yield and quality were assessed using a NanoDrop Spectrophotometer (ThermoFisher, UK) and a Qubit Fluorometer (ThermoFisher, UK) using the Qubit double-stranded DNA High Sensitivity Assay Kit (ThermoFisher, UK).

### Bacterial culture

100 μl of fresh milk from each quarter were spread onto Coliform ChromoSelect agar plates (Sigma Aldrich, UK) to select for growth of coliforms—specifically *Escherichia coli*. The plates were incubated for 18–24 h at 37 °C and the *E. coli* colonies (showing a blue pigment) were counted and recorded. A mean value was calculated using the counts from each quarter per sampling point.

### Quantitative (q)PCR

The number of copies of the 16S rRNA gene were measured to assess bacterial load in the milk samples (Fig. 1). qPCR mastermixes were set up using Brilliant III Ultra-Fast qPCR Mastermix (Agilent Technologies, United States), reference dye (Agilent Technologies, United States) and primers and probes listed in previous work [15]. Each reaction was carried out in triplicate in a final volume of 20 μl, containing 1 μl of extracted DNA which included DNA standards (from 10^5^ to 10^1^ gene copies per μl) and a non-template (or “negative”) control (NTC). Absolute quantification was carried out using a Stratagene MX3005P qPCR System (Agilent Technologies, UK) using the following cycling conditions: 95 °C (5 min), followed by 40 cycles of amplification at 95 °C (15 s) and then 60 °C (30 s). Standard curves were created from the threshold cycle (C_T_) values using the Stratagene MxPro Software (Agilent Technologies, UK). The outputted values were then converted into gene copy number per ml of milk. Wilcoxon signed-rank tests were carried out using the compare_means function in R [16] to assess the effects of time point and treatment group on 16S rRNA gene copy number.

### 16S rRNA gene sequencing and bacterial community analysis

Due to the low biomass nature of milk and the udder, samples and consequent sequences were carefully curated for analysis (Fig. 1). Samples to be submitted for sequencing were selected based on 16S rRNA gene counts by qPCR. Seven samples were not included as they had undetectable 16S rRNA gene counts. The fifty-one remaining DNA extracts, including a mock bacterial community and a non-template control were submitted to Integrated Microbiome Resource (Canada) for sequencing as per the following protocol—https://imr.bio/protocols.html—using custom dual-indexed primers outlined previously [17]. Using the mock bacterial community (20 Strains Even Mix Genomic Material ATCC MSA-2002, ATCC, United States), the mean sequencing error rate was calculated as 0.01%. The raw sequence files are available via the European Nucleotide Archive (ENA) under accession number PRJEB43646.

Prior to analyses, samples with low sequencing depths (< 500 sequences) were removed (n = 17) and the remaining samples (n = 34) were taken forward (Fig. 1). Sequence analysis was carried out using mothur software [18] as described in detail previously [17, 19]. An operational taxonomic unit (OTU)-level (i.e., 97% similarity) analysis generated 1705 unique taxonomic groups. Taxonomic overlap was evident when comparing the non-template control to the milk samples (see discussion), and so removal of these OTUs was deemed inappropriate. A mean of 2818 sequences per sample remained after quality control and chimera removal.

The following steps were carried out using mothur, unless stated otherwise. The Shannon and Inverse Simpson indices were calculated per sample to assess alpha diversity. A distance matrix was compiled using Yue and Clayton theta similarity coefficients [20] with clustering by group visualised by non-metric multidimensional scaling (NMDS). To identify bacterial taxa that were significantly different in relative abundance between groups, Metastats [21] was used, with the P-values adjusted using Bonferroni correction.

### Assessment of exogenous contamination

A non-template control was amplified and sequenced to aid in identification of DNA contamination from laboratory processes. This yielded 224 reads from 25 bacterial families. The majority of these families were not previously reported reagent contaminants [13] but rather taxa that were abundant in the study including Lachnospiraceae, Staphylococcaceae, and Pseudomonadaceae. The relative abundance of these taxa was highly variable between samples and did not hold a consistent profile. Therefore, the authors concluded that their sparse representation in the negative control likely resulted from background barcode bleed in sequencing, and was not sufficient evidence to subtract these taxa from the dataset.

The data was then assessed for artefactual associations between taxa, as a contamination event may be expected to comprise DNA from several species. Reads from the 34 samples taken forward were binned by classification (genus level or equivalent), subsampled, and convert to BIOM format using mothur and biom-format version 2.1.8 [22]. A correlation matrix was produced for the 185 taxonomic groups using FastSpar version 1.0.0 [23] (an implementation of the SparCC algorithm [24]). Heatmaps of clustered correlation scores were generated using R version 4.0.4 [16] and the gplots package [25] (Additional file 1: Fig. 1). The relative abundances of 25 phylotypes were visualised using R and the ggplot2 package [26] (Additional file 2: Fig. 2).

**Fig. 2:**
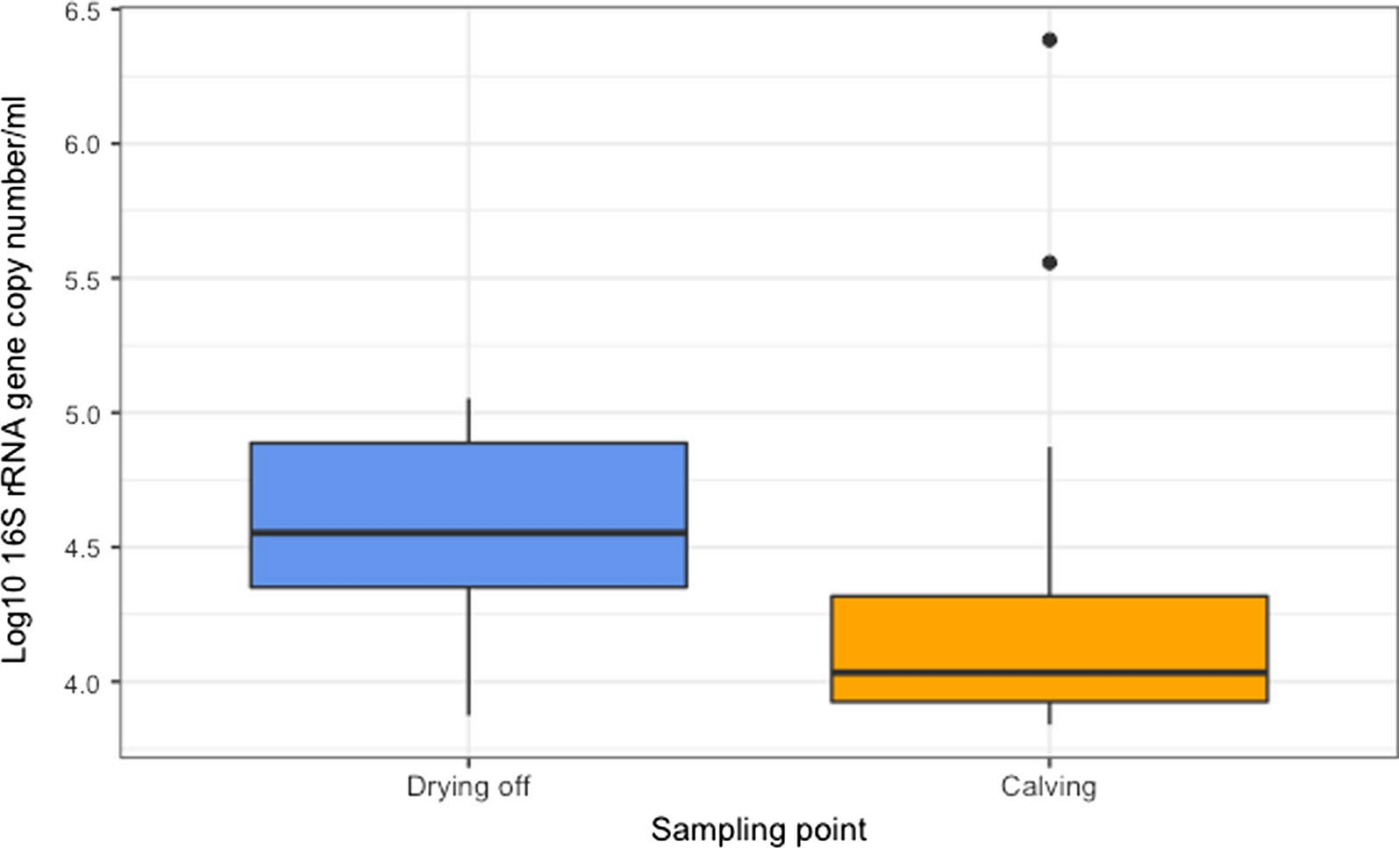
Mean log (10) 16S rRNA gene counts per ml milk at drying off and calving

## Results

### Bacterial load measured by qPCR

Despite differences in copy number of the 16S rRNA gene among bacteria, bacterial load may be broadly inferred by total gene copies. The bacterial load in the pooled milk samples was highly variable between cows and sampling point, ranging from 0 to 2.43 × 10^6^ 16S rRNA gene copies/ml milk. No significant differences were observed when comparing the three treatment groups (P > 0.05). However bacterial load was significantly higher at drying off than at calving (Fig. 2; P = 0.039) and there were no interactions between treatment group and time of sampling (P > 0.05).

### Assessment of exogenous contamination

We aimed to identify patterns of exogenous DNA contamination in addition to the sequenced non-template control. Contamination may impact interpretation, as the presence and abundance of contaminant taxa from a common source are likely to correlate more closely than a true biological signal. FastSpar correlation scores revealed a block of co-associated taxa (Additional file 1: Fig. 1A) supported by P values < 0.05 which could have been introduced to the udder environment together from a common source, such as faeces. These taxa were plausible bovine gut inhabitants and not typical reagent contaminants. However, closer examination suggested that the correlation block was driven by an identical signal in 10 samples (Additional file 2: Fig. 2). These samples were from different cows and collected and processed on different dates, leading the authors to conclude that the pattern was likely the result of inter-sample contamination at a later point in the workflow. Since the correlating phylotypes are present at varying abundance throughout the dataset, the artefact comprises most of the data from the 10 samples, and the source was uncertain, the authors chose to remove these 10 samples from the analysis. The removal of samples due to zero bacterial counts and exogenous contamination negatively impacted the statistical power of the study (Fig. 1), and so a descriptive analysis of the milk microbiota from drying off to calving is carried out hereafter.

### Descriptive analysis of the milk microbiome

In summary, 19 bacterial phyla were found with the most dominant being Proteobacteria (46.1%), Firmicutes (23.5%), Bacteroidetes (21.3%) and Actinobacteria (8.2%). There were 114 bacterial families, with 11 of these comprising more than 1% of the total reads—Pseudomonadaceae (31.6%), Enterobacteriaceae (14.6%), Lachnospiraceae (8.3%), Moraxellaceae (7.8%), Staphylococcaceae (7.7%), Rikenellaceae (3.9%), Propionibacteriaceae (3.8%), Streptococcaceae (3.5%), Ruminococcaceae (2.5%), Corynebacteriaceae (1.6%) and Pasteurellaceae (1.1%).

**Fig. 3:**
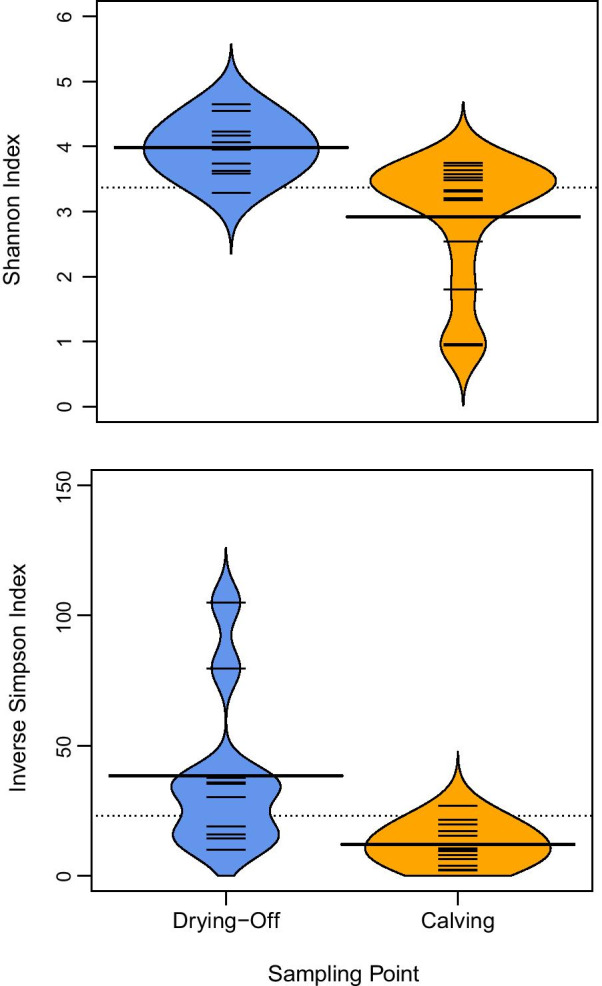
Beanplots showing the range of diversity indices within and between time points in all milk samples

**Fig. 4:**
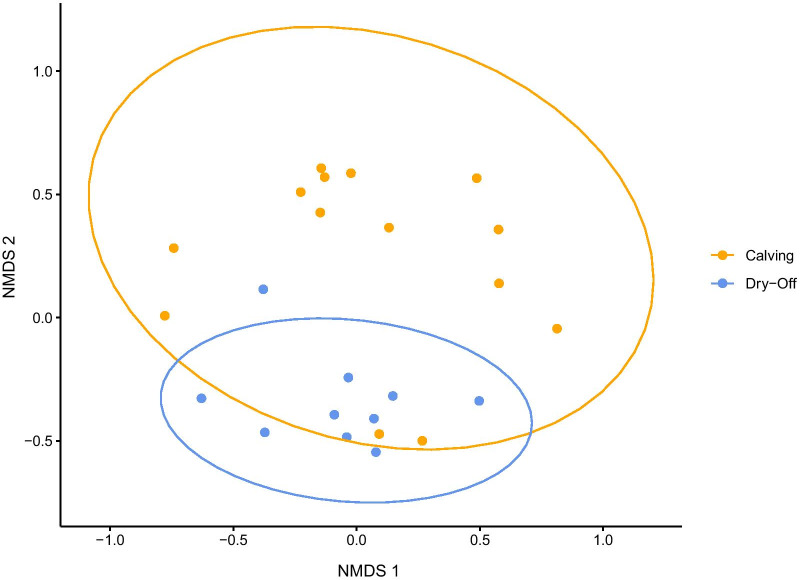
NMDS plots showing distinct clustering when comparing drying off and calving milk samples across all treatments

There were decreases observed in both diversity indices from drying off to calving (Fig. 3). When considering microbiome community structure, clustering by sampling point was observed by NMDS (Fig. 4).

To establish which microbial taxa may have underpinned these changes in community structure by sampling point, comparisons of taxonomic relative abundances were made. There were significant shifts in dominant bacterial taxa between drying off and calving (Table 1), with a decrease in an unclassified Lachnospiraceae, and increases in *Escherichia coli* and *Staphylococcus aureus*. Four samples from the calving timepoint had profiles with decreased diversity, dominated by a single phylotype (*Pseudomonas* at 97.5%, 79.5% and 54.4%, and *Streptococcus* at 55% relative abundance respectively). Two of the *Pseudomonas*-dominated samples also had a large increase (42-fold and 296-fold) in 16S rRNA gene copy estimation between the drying off and calving time points from the same animal, suggesting that there was a concurrent increase in bacterial load.

**Table 1:** Changes in relative abundances of dominant taxa between drying off and calving

Drying off	Calving	Taxa	P-value
0.74 ± 0.33%	1.60 ± 0.04%	*Escherichia coli*	0.050
12.2 ± 0.30%	5.74 ± 0.23%	Unclassified Lachnospiraceae	0.008
18.0 ± 1.10%	15.4 ± 1.83%	*Staphylococcus aureus*	0.036

### Bacterial culture

**Fig. 5:**
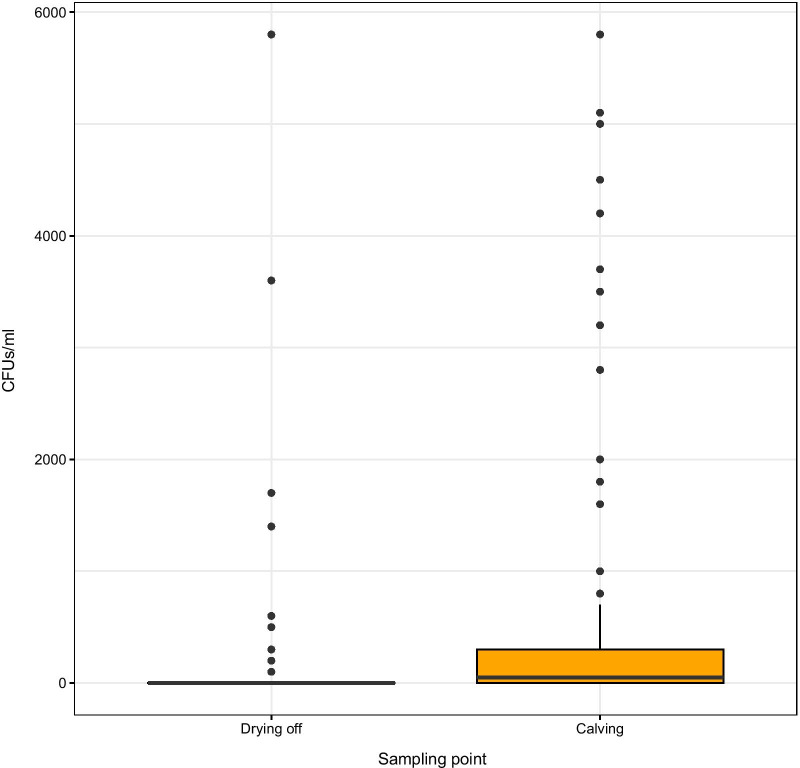
*E.* coli colony counts per ml of milk at drying off and calving

The number of *Escherichia coli* (*E. coli*) colonies per ml of pooled milk was calculated, and comparisons were made between samples taken at drying off and at calving (Fig. 5). The *E. coli* counts were highly variable within and between treatments (i.e. between 0 and 5800 colony forming units (CFU/ml milk), with a significantly higher count being observed in samples at calving (P < 0.0001).

## Discussion

This animal experiment was designed to study milk microbiota compositional shifts before and after calving, across differing drying off treatment protocols. Despite careful handling of samples throughout processing (discussed in detail below), low biomass milk samples and contamination had a profound effect on the sample numbers for the microbiome analysis. As such, statistical assessment of treatment effects was not possible as part of this work. Nevertheless, we have critically profiled the milk microbiome from samples before and after drying off and carried out a descriptive analysis to inform future larger-scale studies to test specific hypotheses.

### The challenges of studying low biomass communities

In samples with a low biomass, it is critical to address the potential impacts of contamination on microbiome datasets. We recruited cows that did not have signs of clinical mastitis at drying off, which means that the bacterial load was likely much less than that of cows with mastitis. As such, we believe our data set is more sensitive to contamination effects. The following considerations were made, and steps to minimise these effects were carried out.

First, we measured the bacterial load in each pooled milk sample by qPCR, which was used as a selection criterion for submitting samples for 16S rRNA gene sequencing. We excluded samples which had undetectable or zero 16S rRNA gene copies. The majority of samples taken forward had over 1 × 10^4^ 16S rRNA gene copies/ml, which appears to be a crucial tipping point, as two previous studies have shown that samples with less than 1 × 10^4^ bacterial cells/ml are more sensitive to contamination effects [13, 27].

Second, we considered removal of OTUs that were present in our non-template control (NTC). A relatively low sequencing coverage was observed in these milk samples, compared to higher biomass samples such as faeces. Consequently, contaminating taxa from laboratory reagents can have a profound impact on microbiome data, but whether these should be removed is still under debate, as there may be overlapping taxa which can genuinely be found in samples [12]. We only obtained 224 reads from the NTC, with low read numbers representing many taxa. The most encountered NTC taxa in our dataset were *Cutibacterium* species, unclassified Micrococcales, *Staphylococcus* species, unclassified Lachnospiraceae, *Pelomonas* species and *Pseudomonas* species. However, many contaminating genera such as *Corynebacterium*, *Pseudomonas*, *Staphylococcus*, *Propionibacterium* and *Streptococcus* species are also known colonisers of the udder environment [28]. The taxa present in the NTCs did not significantly correlate with one another according to FastSpar analysis, suggesting that they were not co-introduced from a common source. Therefore, we did not it deem it appropriate to remove any OTUs from our dataset, as key microbiome shifts may have been missed. To minimise contamination effects, we studied core and dominant OTUs which were present across samples at a minimum level of 1% relative abundance, which are less likely to be influenced by contaminants.

Third, we cultured a dominant genus to provide evidence that the sequencing data is not only composed of PCR and sequencing artefacts. We selectively cultured *E. coli* from all milk samples, with a range of 0 to 5800 cells/ml of whole milk being observed. The culture results broadly reflect the relative abundance of the *Shigella/Escherichia* phylotype in the microbiota analysis.

Fourth, we randomised DNA extractions and carried these out in small batches to minimise batch effects. Salter et al. [13] compared nasopharyngeal microbiome samples from children at two different time points, and although the samples clustered separately, this effect was due to bias driven by contamination from the DNA extraction kits used. Randomisation of samples prior to extraction has been suggested previously [12]. Nonetheless, rhizosphere organisms (often detected as contaminants) are present at low levels in our dataset, as indicated by the non-template control, and we also detected patterns of exogenous DNA contamination as part of this study. The taxa observed included likely gut inhabitants and not typical reagent contaminants, but a more detailed examination showed a correlation block that was driven by an implausibly identical signal in 10 samples. Since the source of this contamination was uncertain, we chose to eliminate these samples from the analysis.

### Study design limitations

Although we carried out these analyses as carefully and critically as possible, we do recognise that there are limitations to this study design. Specifically, a marked decrease in sample numbers per treatment group occurred due to our filtration steps, meaning that many of our statistical analyses on the microbiome dataset could not be carried out. Although a larger study would have provided a deeper insight into multifactorial effects on the milk microbiome, it was not possible to increase the scale of this experiment. Small and well-designed studies defining a population of interest have the potential to advance knowledge in the field [29], but we did not expect this magnitude of sample loss. Given the potential for contaminants to significantly affect the results in 16S rRNA gene sequencing studies, we recommend considering similar steps as presented here in future work and to anticipate marked decreases in sample numbers.

### Bacterial load and *E. coli* counts

We found that bacterial load decreased after drying off, which appeared to be independent of treatment protocol. Interestingly, previous work has shown the opposite effect [6]. It has been proposed that the use of internal teat sealant as a physical barrier alone may be effective in reducing bacterial colonisation [6], which would explain the overall decrease in bacterial load and gut- associated taxa specifically, that we observed as part of this study. In the presence of a reduced overall bacterial load, *E. coli* counts by culture increased—suggesting that after the transient introduction of bacteria from the external environment diminishes, some species can thrive in this niche.

We found that the presence of cloxacillin, a narrow-range antimicrobial, did not have a significant effect on bacterial load after treatment. It is challenging to make comparisons to other work, since different antimicrobial classes can be used at drying off. Nonetheless, previous work has shown that ceftiofur (a broad-spectrum antimicrobial agent) treatment at drying off had no impact on bacterial load by qPCR [6, 30]. Indeed, other work has highlighted that the number of new occurrences of mastitis during dry cow therapy was not different when comparing cows administered antimicrobials or teat sealant only [30–33]. Therefore, it may not be surprising that significant differences in bacterial load after antimicrobial treatment were not observed in our dataset.

### Microbiome changes by sampling point

Previous work has shown that the milk microbiome changed dramatically over the drying off period [6, 34], with sampling point being the key influencing factor rather than antimicrobial treatment. We showed that when bacterial colonisation decreased, the richness and evenness in bacterial communities decreased, which has been shown previously by Derakhshani et al.[34]. Many studies have highlighted that a decrease in microbiome alpha diversity is linked to cattle with mastitis, with pathogens becoming more dominant after drying off being a risk factor for the development of mastitis during lactation [9, 30, 35].

Most samples in this study revealed a diverse microbiota, largely composed of gut-associated and skin-associated taxa. The presence of these bacteria in milk samples is likely to reflect the vulnerable nature of the teat in dairy cattle, where impaired teat sphincter function common in mechanically milked cows [36, 37] permits bacterial access to the teat canal from the environment. In keeping with this hypothesis, the bacterial load and proportional abundance of many gut-associated taxa was somewhat lower at calving—after prolonged sealing of the teat—than at the first timepoint. This diverse assortment of bacteria is utterly distinct from a typical infection profile involving a single organism. The cows with milk samples dominated by *Pseudomonas* and with a high bacterial load did not develop mastitis, but their microbial profile suggests that a subclinical infection may have been establishing itself by the calving timepoint.

Between drying off and calving, there was a reduction in unclassified Lachnospiraceae, which is a gut-associated family likely to be decreased because of the physical barrier imparted by the teat sealant. *E. coli* counts by both 16S rRNA gene sequencing and culture increased between drying off and calving, with *Staphylococcus aureus* being a dominant member of the microbiome across both time points—both of which are potential causative agents of mastitis.

## Conclusions

We found that the drying off process changed bacterial load and microbiome composition—with these factors appearing not to be affected by the presence of an intramammary antimicrobial agent. Milk microbial communities are challenging to study, and we have provided a critical analysis to ensure that contamination effects were minimised. This study sets the scene for further work to fully characterise the milk microbiome in the unique setting of modern dairy farms, and to establish whether different thresholds could be used in future for treatment selection at drying off.

## Supplementary Information

**Additional file 1: Fig. 1.** Heatmap of pairwise correlation scores for 185 genus-level phylotypes derived from 34 samples with FastSpar. No significant phylotype correlation was found for most taxa. A small number of abundant phylotypes are highly correlated (0.25–0.75, p < 0.05) in two groups: (A) probable gut-associated bacteria (including Bacteroides and unclassified Clostridiales, Lachnospiraceae, Muribaculaceae, Prevotellaceae, Ruminococcaceae) from a contamination artefact, and (B) probable skin-associated bacteria (including *Actinobacteria*, *Cutibacterium*, *Staphylococcus*) present throughout the dataset.

**Additional file 2: Fig. 2.** Proportional abundance profiles of the 25 most abundant phylotypes in the dataset, including those from the highly correlated groups A and B (see Additional file 1: Fig. 1). The inter-sample contamination artefact can be seen in the ten samples on the right which were discarded from further analysis.

## Data Availability

The generated raw 16S rRNA gene sequence fastq files (with primers removed) are available publicly through the European Nucleotide Archive (ENA) under accession number PRJEB43646. All data analysed during this study are described in the manuscript, with statistical outputs and sample metadata being made available in the supplementary information files and within the ENA submission.

## Abbreviations

16S rRNA: 16S ribosomal ribonucleic acid
AMOVA: Analysis of molecular variance
*E. coli*: *Escherichia coli*
ISI: Inverse Simpson index
NMDS: Non-metric multidimensional scaling
NTC: Non-template control
OTU: Operational taxonomic unit
SCC: Somatic cell count
qPCR: Quantitative polymerase chain reaction
