# Supplementary figures and images for: Milk microbiome in dairy cattle and the challenges of low microbial biomass and exogenous contamination
Source: Anim Microbiome. 2021 Nov 18;3:80. doi: 10.1186/s42523-021-00144-x (PMC8600933; doi:10.1186/s42523-021-00144-x)

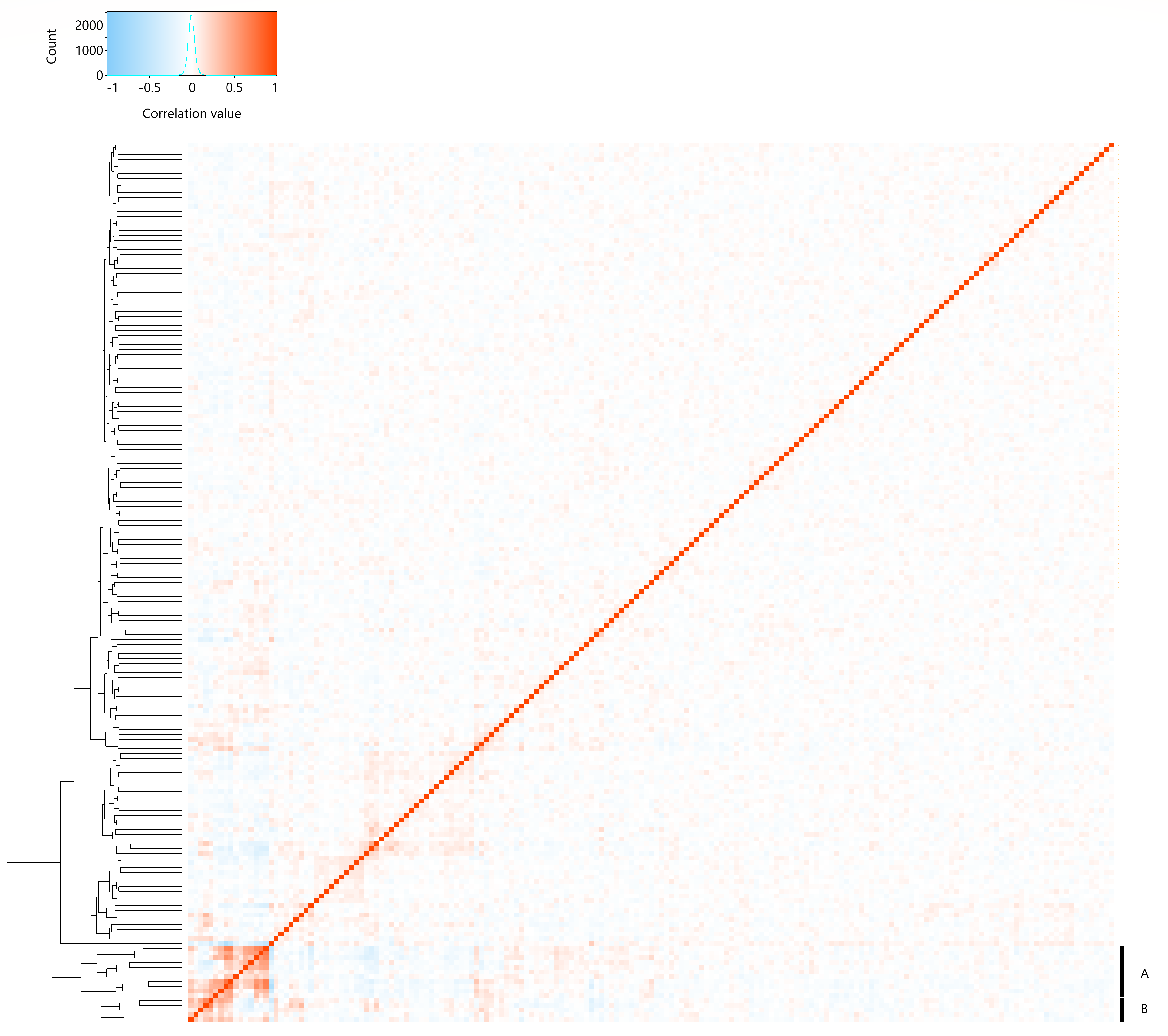

Supplement: Supplementary file 1 — Additional file 1: Fig. 1. Heatmap of pairwise correlation scores for 185 genus-level phylotypes derived from 34 samples with FastSpar. No significant phylotype correlation was found for most taxa. A small number of abundant phylotypes are highly correlated (0.25–0.75, p < 0.05) in two groups: (A) probable gut-associated bacteria (including Bacteroides and unclassified Clostridiales, Lachnospiraceae, Muribaculaceae, Prevotellaceae, Ruminococcaceae) from a contamination artefact, and (B) probable skin-associated bacteria (including Actinobacteria, Cutibacterium, Staphylococcus) present throughout the dataset. [file 42523_2021_144_MOESM1_ESM.png]

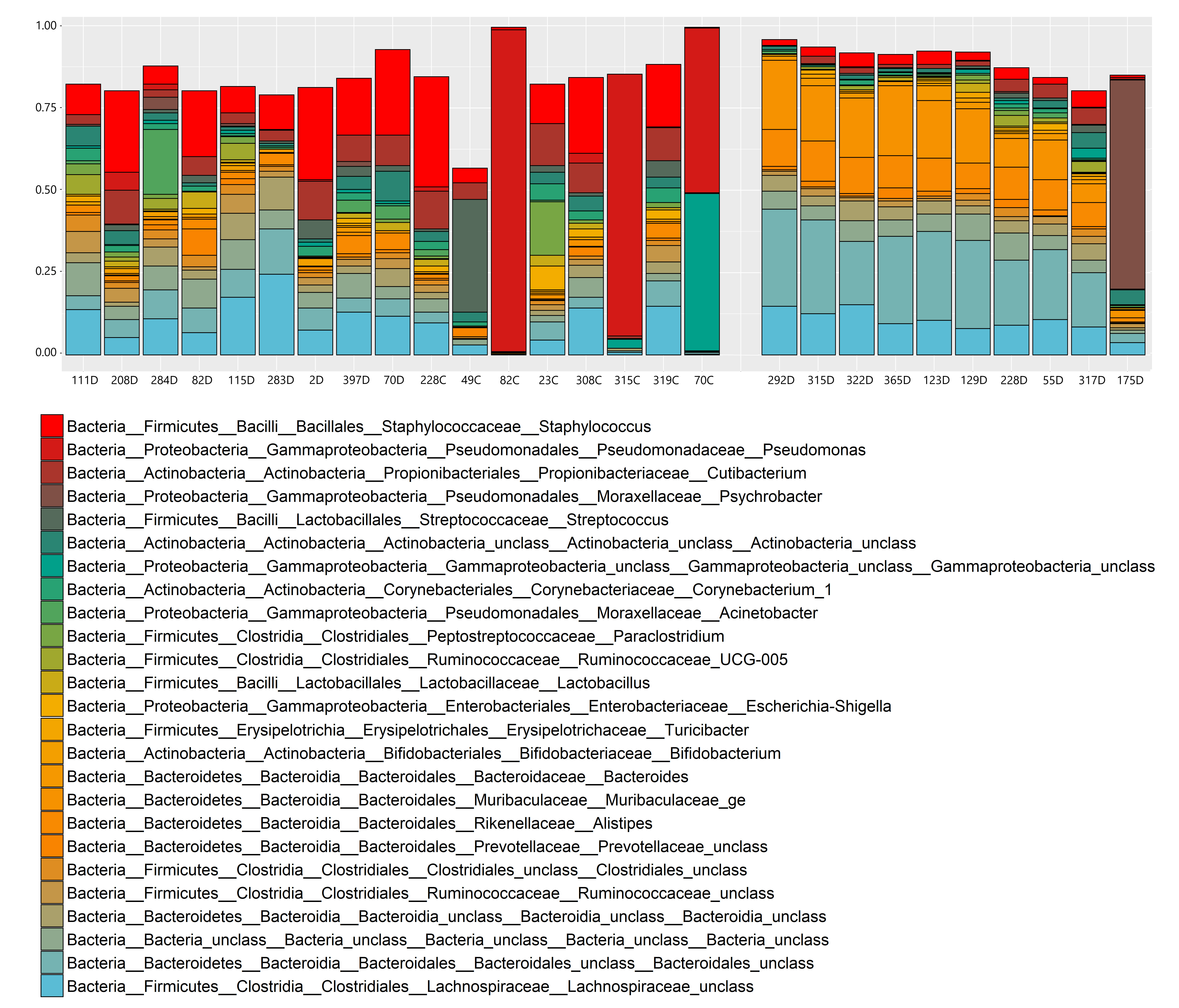

Supplement: Supplementary file 2 — Additional file 2: Fig. 2. Proportional abundance profiles of the 25 most abundant phylotypes in the dataset, including those from the highly correlated groups A and B (see Additional file 1: Fig. 1). The inter-sample contamination artefact can be seen in the ten samples on the right which were discarded from further analysis. [file 42523_2021_144_MOESM2_ESM.png]
